# Subjective position sliding in utterances perceived as echolalic in a child with Language Disorder: a case study

**DOI:** 10.1590/2317-1782/20232022258en

**Published:** 2023-12-18

**Authors:** Juliana Bonatto, Natália Faloni Coelho, Lourenço Chacon

**Affiliations:** 1 Programa de Pós-graduação em Fonoaudiologia, Universidade Estadual Paulista - UNESP - Marília (SP), Brasil.; 2 Programa de Pós-graduação em Estudos Linguísticos, Universidade Estadual Paulista - UNESP - São José do Rio Preto (SP), Brasil.; 3 Departamento de Fonoaudiologia, Faculdade de Filosofia e Ciências, Universidade Estadual Paulista - UNESP - Marília (SP), Brasil.

**Keywords:** Child Language, Language Disorder, Echolalia, Linguistics, Speech Therapy

## Abstract

Within a linguistic-discursive framework, subject markers in a chain of utterances considered to be echolalia based on the recurring linguistic structure *does X want Y?* were investigated. This chain was produced during a speech therapy session by J., a female child, 10-years-old at the time of data collection, and with a speech-language pathology diagnosis of language disorder and a medical diagnosis of early psychosis. A set of linguistic fluctuations indicated a sliding of the subject position in the analyzed chain. Such fluctuations involved syntactic, lexical, semantic, morphological and prosodic elements. Discursively, the fluctuations left traces of a sliding of the subject position in the chain formed by these utterances, from a *spoken* subject (*do you want Y?*) to a *speaking*/*desiring* one (*I want Y*.). In this way, utterances considered echolalia can provide clues, via their linguistic fluctuations and discursive slippages, about the subject's desire in their relationship with the O/other. Given this, although they do not emerge in a conventional way, such utterances can demonstrate possibilities for changes in subject position. A contribution of the present research for clinical practice involving language in therapeutic settings therefore, was to highlight a listening to utterances, which could be seen as connected/grounded in the speech of the other. In clinical practice involving language, it is possible to create space for new/other senses for utterances, to allow the constitution of the subject of/in language, based on utterances often interpreted as being devoid of subjectivity.

## INTRODUCTION

In pathological contexts involving language disorders, so-called echolalias were initially viewed as “[…] productions devoid of sense, only an echo, similar to the speech of a parrot: a repetition without communicative intent, without interlocutors […] simply a decontextualized empty repetition, […]”^(1:416)^. They have subsequently begun to be investigated through different approaches^(2)^.

Approaches related to pragmatic, communicative or social interaction aspects have predominated^(3-5)^. However, based on a multimodal and enunciative language approach, echolalias can also be understood as a metaphor, in their relation to gestures in autism^(1,6)^. However, they can also be understood, based on the subject/language relationship, as repetitions of sonorous segments that lead to changes in verbalization^(7)^.

Therefore, based on this prior case, we can see that only more recently we encounter in its investigation, a focus on the *subject*, who beyond the echolalias, “speaks in this voice”^(2:2)^, a perspective that produces a significant change of direction in the trajectory of the investigation of speech understood as echolalia.

Adopting this latter perspective and distancing ourselves from the theoretical-methodological understanding that views echolalias as echoes of the other(s), we seek to investigate how this speech could be interpreted from the linguistic-discursive point of view of a French orientation, whose characteristics will be highlighted subsequently. Within this framework, possible subject markers were sought in utterances with recurring linguistic structures in the linguistic-discursive production of J. - a child with a speech therapy diagnosis of *language disorder* and a medical diagnosis of *early psychosis.*

In the expression *linguistic-discursive*, “linguistic” is understood as a structural organization of the different dimensions of language. In this organization, the syntactic, semantic, morphological and phonological (in its prosodic component) dimensions have been privileged in speech understood as echolalia. In this same expression, “discursive” is understood as a process of sense production, “[…] whose specificity resides in the type of materiality of its base, that is, linguistic materiality […].”^(8:179)^, materiality that manifests itself in the form of utterances. However, we also highlight that the prosodic component of the phonological dimension of language, in addition to maintaining a strict relationship with the syntactic and semantic components of language, also indicates subjective aspects of the discursive process.

What we understand here as an utterance is not a structural unit of language. In other words, the utterance is not confused with the syntactic organization of a sentence or clause; it is a concrete and singular materialization of language in a discursive process. In this process, it arises as “[…] a link in a very complex chain of other utterances […]”^(9:291)^ - which means that an utterance does not begin in itself, given that it is anchored in a network of already produced utterances.

In summary, the conception of the subject adopted in our proposal is that he/she is constituted by the O/other, *in*/*through* *language* ^(8,10,11)^. From this perspective, the subject is not the empirical subject, given that they are not understood as the origin of (their) speech, but as its substrate and effect^(10)^. Further “[…] they are always simultaneously, the subject of ideology and the unconscious […] traversed by language prior to cognition”^(12:188-189)^.

In the *Other* (ideology and the unconscious) is the position “[…] of the truth regarding the symptom and desire”^(13)^. As such, the subject’s desire marked in their discursive process by sliding, will be considered in detail in the present study. It is notable however, that in a discursive process, the Other is linguistically marked. We are dealing in this case, with that instance that we can understand as the “other”, that is, the individual who, interpellated as subject by the language in the discursive process, is configured as a linguistically marked interlocutor in this process. In summary, the “other” corresponds to the alterity that is, in some way, revealed in the discursive chain.

Among the linguistic-discursive characteristics that justify our interest in the subject markers present in utterances with a recurring linguistic structure produced by the child being considered, is the fact that the linguistic markers most commonly observed in children without language acquisition difficulties that indicate subjective constitution were not observed here: the first-person pronouns such as *I, my, me, mine, with me*. As such, in the utterances that one expects as declaratives of J., a prevalence of second person pronouns (instead of first person) in the interrogative form was observed. Therefore, utterances that we would generally expect to appear in J.’s speech such as *I want to go*, *I want to sleep*, *I want water* appear as *do you want to go?* *Do you want to sleep*? *Do you want water?* In the last instance, the conventional and expected form in first person of her utterances is displaced to third person - such as *Does the girl want cake?* instead of *I want cake*.

In these utterances, their recurring structure in syntactic terms is *does X want Y*. In this structure, both the first and third elements can either be missing or undergo lexical variation during speech production. The following linguistic characteristics, however, remain constant: semantic, volitional in the verb *to want*; morphological, in the present indicative of this verb; and prosodic, in the interrogative intonational form of the utterance. Given the repeated presence of this linguistic form in J.’s utterances, we ask: is it possible to discursively observe traces of subject position sliding, that is, from the *spoken* subject (*do you want Y?*) to a *speaking*/*desiring* one (*I want Y.*) in the chain formed by these utterances? If so, how does such a sliding manifest?

The hypothesis associated with such a question is that J.’s chain of utterances, presenting this recurring linguistic structure (traditionally called *echolalias*) would make up a sequence in which they are not simply “echoes” of the other’s speech, but a manifestation/marker of subjectivity in discursive production.

Notably, two studies have focused on the speech production of J., seeking, similar to here, the linguistic-discursive markers of her subjectivity. In one of these^(14)^, the hesitations were viewed as such markers; in the other^(15)^, it was the markers of refusal that were understood in this manner. However, we believe that utterances with recurring linguistic structures, similar to those that constitute the object of the present investigation, can provide other (and different) indicators of subjectivity in J.’s speech, increasing our understanding of how, in a non-conventional manner, subjectivity can be observed in clinical cases such as J.’s.

## PRESENTATION OF THE CLINICAL CASE

The present study was developed according to the following ethical procedures: a review by the Research Ethics Commission of the Faculty of Philosophy and Science of the São Paulo State University (Faculdade de Filosofia e Ciências/Universidade Estadual Paulista - FFC/UNESP), number 0138/2010; and signing of a Free and Informed Consent Form (ICF) by the child’s legal guardian, using the model provided by the Centre for Studies in Education and Health of the Faculty of Philosophy and Sciences of the São Paulo State University (CEES/UNESP).

Filmed and transcribed records of a speech therapy session of around 40 minutes were used, with a child (identified in the data as J.) of female sex, 10 years old at the time of data collection. The session underwent three transcription phases: (i) by a first transcriber; (ii) a revision of the transcription by the same transcriber and by the supervisor monitoring the child’s clinical progress; and finally, (iii) a third reviser, who specifically added information to the transcription regarding the immediate situation in which the therapy session was undertaken. Such information included gestures, facial expressions and basic prosodic information. The final transcription was prepared according to the norms proposed for the Research Project of the São Paulo Study of Urban Linguistic Norms (Project NURC/SP), which studies spoken Portuguese. According to these norms: **+** corresponds to a silent pause; **?** corresponds to interrogative intonation; **(( ))** corresponds to observations by the transcriber; **:** corresponds to elongations; **/** corresponds to interruptions; **( )** corresponds to moments of speech unintelligible for the transcriber or uncertainties in the transcription; and finally, **[ ]** corresponds to overlapping voices.

The child was given a speech therapy diagnosis of *language disorder* and a medical diagnosis of *early psychosis*. At the time of data collection, she had already been undergoing speech therapy for three and a half years. In carrying out therapy, grounded in a clinical framework with a pragmatic perspective, the sessions sought to “restructure” the child’s linguistic expression. From this perspective, strategies such as play activities were employed, that involve “pretending” games that simulate conventional, day-to-day situations in which language is involved. This is the case of the session under consideration here based on a “pretending” game of giving a baby a bath.

Regarding the speech therapy diagnosis of *Language Disorder*, beyond the alterations at the formal language level, J. mainly presented changes in discursive aspects of language. Regarding these, the I/(O)other relationship showed itself to be particularly weakened, which could be observed in her discursive production, by: (i) an absence of the first-person pronoun; (ii) dispersion of the syntagmatic chain; and (iii) a return of utterances (not necessarily present in the therapeutic scene). Among these utterances, those previously presented, with a recurring linguistic structure of the type *does X want Y?,* stood out. Therefore, given this prominence, it is in/through the irruption of these utterances that the subject markers manifest in them, were perceived.

## DISCUSSION

Here, we outline the investigation’s guiding questions: is it possible to discursively observe signs of the sliding of the subject position, that is, from the *spoken* subject (*Do you want Y?*) to the *speaking*/*desiring* subject (*I want Y.*) in the chain formed by these utterances? If so, how does the sliding manifest?

Notably, in the structure *does X want Y?*, the first and third elements can be missing. When filled in, in position X, fluctuations between the second and third person can occur, with a predominance of second person. In position Y, verbal elements can occur (such as *to sleep* or *to write*) or nominal elements such as (*water*). As we can see, the elements that can occupy the positions X and Y could either be missing or vary, while the (i) verbal volitional element *to want* and (ii) the interrogative intonation of the utterance, can remain constant.

Chart 1 presents a sample of the therapy session that will be analyzed, during which, mainly utterances with this structure arose. Such utterances are highlighted in bold in the chart.

**Chart 1:** Sample of a therapy session with different irruptions of utterances with the structure *does X want Y*?

037	**J**	**Do you want to sleep?**	J places the pillow on the bed, and then speaks while looking at T.
038	T	That’s right ... I won’t go to sleep now ... will you go to sleep now?	T arranges the pillow on the bed and then J pushes the bed to the side.
039	J	((laughs))	J pushes the bed against the wall.
040	T	I won’t go to bed now, no	J and T look at each other.
041	J	sit ... you can sit	J goes over to the toy bed, and after looking at T, places one of her hands on the bed, as if she was pointing at it.
042	T	Put the baby down to sleep ... put it down	J looks at T, while she picks up the doll that was on the floor.
043	J	((laughs))	J goes over to the bed.
044	T	ah ...you want to lie on the bed ... lie here ... lie here for the baby ... ah ... there isn’t enough room for you there	T, who is still holding onto the doll, points at the mattress, while looking at J. J threatens to support herself on the bed using her arms.
045	J	She is sleeping	J says, looking at T.
046	T	Who is sleeping?	J looks at the ceiling.
047	**J**	**(do you want some water?)**	J and T look at each other.
048	T	The baby is here ... with me	T shows the doll in their hands.
049	**J**	**(do you want some water?)**	J looks down.
050	T	The baby is here with me	J picks up the toy that was on the floor that belongs to the bathroom kit.
051	**J**	**Do you want to write?**	J says, looking at T, who keeps observing the doll.
052	T	No we’re not going to write today ... today I didn’t bring any paper:: to write:: ... or a pen::cil	J keeps looking at T for some time before looking down again. T speaks while J starts to take the clothes off the doll.
053	**J**	Do you **want some water?**	J looks at T, while she keeps taking the clothes off the doll.
054	T	I’m not thirs::ty	J looks at T, while she continues taking the clothes off the doll.
055	**J**	(the baby did) ... {the baby did a poo	J says looking at T and points at the baby
056	T	{will you have a bath? ... did a poo? ... see if it’s smelly	J. keeps looking at T who, after smelling the doll, offers it to J.
057	J	((J cries out))	J takes the doll out of T’s hands.
058	T	ahn it’s smell::ly	J places the doll on the bed.
059	J	**I’m going to go to sleep Mila ... what are you doing?**	J takes the sheet off the bed.
060	**T**	It’s smelly ... so let’s give it a bath ... if the baby did a poo	J spreads the sheet over the mattress that is behind her.

The first appearance of an utterance with the structure in question can be observed in the table, that is: *do you want to sleep?* (utterance 37). It initiates a chain of utterances in which the fluctuations of linguistic elements in the positions that accompany the verb allow us to detect the sliding of the subject position. The scene under analysis unfolded around the discursive object making *the baby go to sleep*. Therefore, in general, it could have favoured the emergence of this utterance. The non-verbal elements could be at the root of such an irruption, given that a small bed and pillow were present in the scene.

The participation of interlocutor T. also stood out (the linguistically marked *other* in this discursive process) in the irruption of the chain of utterances presenting the linguistic structure under analysis. Therefore, such an utterance, which could have been, on the part of T, heard as *I [J.] want to sleep*, was heard as *T., do you want to sleep?*, wherein J. receives as a reply from T.: *yes … I won’t go to sleep now … will you go to sleep now?*. Given this “expression of her desire” not being heard, J. responds with a laugh and steps away from the bed. T., in turn, continues not to hear, suggesting that J. put the doll to bed, probably trying to operationalize the therapeutic plan - the pretend play of routine care of a baby - within the clinical-theoretical framework that underpins its realization, the approach called pragmatic.

Not hearing J.’s “expression of desire”, together with the contextual elements of the discursive process, could be at the root of the irruption of another of the utterances with the structure being analysed here: *do you want water?* (utterance 47). In this utterance, the elimination of the position *X* and a lexical fluctuation on the position *Y,* from *to sleep* (a verb) to *water* (a noun), occurs. Another utterance irrupts soon after the utterance *do you want water?* (utterance 49). The slippage in discursive production between, *do you want to go to sleep?* and *do you want water?* could have occurred, once again, due to another failure on the part of T. to listen to J.’s “expression of desire”. Additionally, we once again observe T. in the scene drawing attention to the doll, given their not noticing that the utterance *do you want water?* could be contextually linked to care of the doll. Therefore, the sliding between the two utterances could also have occurred due to the non-verbal elements of the therapeutic scene. A toy bathtub and bathroom kit were part of the scene. It was notable that soon after the new utterance *do you want water?*, J. picks up a toy belonging to the bathroom kit off the floor, an action that suggests an association between this utterance and the toy bathtub present in the physical space of the therapy session - that is, in linguistic terms, a marker of semantic association between *bathtub* and *water*.

The ongoing failure to hear J.’s “expression of desire” means that a new utterance with the same structure irrupts in the chain: *do you want to write?* (utterance 51). This utterance by J. receives the following response from T.: *no, we won’t write today … today I didn’t bring any paper:: to write:: … nor a pen::cil.* A new fluctuation in the lexical element that fills the Y position can be observed: from *water* (a noun) to *to write* (a verb). Notably, in the physical space where the scene took place, there was a small table and child’s stool, non-verbal elements that would have stimulated a connection with writing activities, such as those undertaken in a school environment.

Once again, a failure to hear on the part of T. seems to provoke a further irruption of an utterance with the same structure - *do you want some water?* (utterance 53) -, equally with a fluctuation in the lexical filling of the position Y, that is, of *to write* (a verb) to *water* (a noun). Once again, it is an utterance that irrupts in the discursive process as one more unheard “expression of desire” of (her) place.

Finally, after a series of utterances (53 to 58) in which the discursive object *making the baby go to sleep* shows itself in conflict with the object *give the baby a bath*, an utterance irrupts in J.’s speech, which, although discursively connected to the chain already underway, breaks the linguistic structure that underpinned the utterances constituting this chain: *I’ll go to sleep Mila … what are you doing?* It is the moment when, linguistically and discursively, the sliding of the subject position reveals itself: from the *spoken* subject (by the other) *you* to the *speaking*/*desiring* subject (of themself) *I*. The following elements further highlight this sliding: (i) the lexical fluctuation between the verb *to want* and the verbal locution *to go to sleep*; (ii) the fluctuation of the morphological characteristics of the verb (present) and the locution (future); and, finally, (iii) the fluctuation of its semantic characteristics (respectively, from the volitional to affirmative).

Some observations should be made regarding the irruption of utterances with the linguistic structure *does X want Y?* highlighted above. The progress of the child’s clinical case showed that utterances with this structure occurred in all the therapeutic scenes observed. It is also notable that such utterances irrupt even in therapeutic scenes where no physical object present would apparently explain their irruption. Therefore, the emergence of utterances with the structure *does X want Y?* can be instigated by both verbal and non-verbal elements that echo in the scene and from the already-said/already-experienced background that anchored it. Or possibly and mainly, through the encounter between what was underway in the scene under analysis and echoes from other scenes (not only therapeutic) that make up J.’s discursive memory.

It is evident therefore, that an utterance does not begin in itself, but emerges as a link in a chain of utterances^(9)^ in a discursive process. Ultimately, a discursive process does not begin in itself, given that it establishes itself over a prior discourse (or inter-discourse), made up, in the case under analysis, not only of the set of therapeutic scenes between J. and T. but also, of the multiple scenes (non-therapeutic) that J. was invited to participate in her daily life. Therefore, the utterances that emerged in the scene under consideration would be “[…] strictly speaking, an effect of the interdiscourse over itself […].”^(8:167)^, which reinforces the fact that, as we anticipated, the subject is not the source of (his/her) discourse, but its substrate and effect^(10)^.

Through the fluctuations between linguistic elements that make up the structure *does* *X want Y?,* in some manner related to verbal and non-verbal elements of the therapeutic scene and, as we just indicated, also possibly to discursive memory, the chain of utterances manifested the sliding of J.’s subject position. This sliding did not occur in a linear/progressive manner, as we saw in the description of the sample. By contrast, the lexical fluctuations reflected: (i) developments not favoured in the discursive process due to (very) little negotiation of sense between T. and J.; (ii) conflicts between discursive objects; and, finally, (iii) movements that combined echoes of other utterances from the scene, and non-verbal elements that integrated it. These are fluctuations that “construct” the desired discursive sliding - which, from the first to the final utterance, we can linguistically describe through displacements: lexical (*to want*/*to go to sleep*); semantic (volitional/affirmative); morphological (present/future); prosodic (interrogative/declarative); and pronominal (I/you). In this context, semantic and pronominal displacements especially reveal a possible sign of the subject’s desire, marked in its extreme positions, by the affirmative and volitional character of the verbs, and by the change of grammatical person, that is, the linguistic and conventional expression of subjectivity.

We see this in *I will go to sleep Mila*, in which the first-person markers point towards the subjectification of J. towards the position of a *speaking*/*desiring* subject, and the continuation of the utterance … *what are you doing?* allows us to identify a possible distance of the child in relation to their own utterance - which concomitantly, can provide another indicator of their change of subject position. Therefore, when viewed within a linguistic-discursive framework such as that adopted in our study, utterances understood as echolalias can demonstrate, through their supposed structural repeatability (but not discursive), a series of displacements between subject positions.

## FINAL CONSIDERATIONS

We believe that the investigation presented here has presented elements for the understanding that a chain of utterances understood as echolalias (given their recurring linguistic structure) can, at the same time, show traces of being anchored (i) in contextual elements from the therapeutic scene, and (ii) in an interdiscursive network that makes the chain possible. In this anchoring, the chain can further reveal indicators of subjectivity, if the irruption of these utterances is understood as an “expression of desire” wherein displacements of the subject position become visible.

However, the functioning of the *does X want Y?* structure during the therapy sessions of the child J., was found to be quite complex, the reason for our proposal of investigating it. As we can see, the linguistic fluctuations in the chain of utterances with a recurring linguistic structure showed that “[…] waywardness [is] always ready to implant itself in discourse, as a result of weakly constituted subjectivity […].”^(15:9)^ However, the movement of the waywardness observed here can indicate a sliding of subject position during discursive production, given that discursive production is a “[…] logically destabilizing site, marked by the tension between the spoken and the unspoken, […]”^(13:27)^.

We believe that the present study can provide contributions for the literature and for clinical speech therapy that focuses on aspects of language. As we sought to show, recurring linguistic structures can, in the utterances that contain them, provide clues through linguistic fluctuations and discursive slippages, regarding the subject’s desire in their relation to the O/other. Therefore, although they do not irrupt in a conventional manner, such slippages can show the possibility of changes to subject position. Given this, a contribution to clinical practice involving language offered by an investigation of utterances, anchored in recurring linguistic structures can be observed, to the extent that, in the therapeutic setting, there can be a listening to utterances that, in the first instance, could be understood as connected/rooted in the speech of the other. In clinical practice with language, it is possible to create space for new/alternative senses for such utterances, so as to favour the constitution of the subject of/in language, based on utterances frequently interpreted as being devoid of subjectivity.

Therefore, in cases of children with language disorders of an implied psychotic nature, speech therapy can go beyond structural/formal analyses and reach a significant result as such (verbal and non-verbal). As we sought to demonstrate, a linguistic-discursive perspective such as that outlined here, rather than analysing expressions in a decontextualized manner with structures that could be interpreted as echolalias, allows us to analyse them in relation to the complexity and nuance of their emergence during the discursive process.
