# Supplementary material for: Subjective position sliding in utterances perceived as echolalic in a child with Language Disorder: a case study
Source: Codas. 2023 Dec 18;36(2):e20220258. doi: 10.1590/2317-1782/20232022258en (PMC10750891; doi:10.1590/2317-1782/20232022258en)
Supplement: Translation

# Translation: Deslizamentos de posição subjetiva em enunciados tidos como ecolálicos de uma criança com Distúrbio de Linguagem: um estudo de caso

**DOI:** 10.1590/2317-1782/20232022258pt

**Authors:** Juliana Bonatto, Natália Faloni Coelho, Lourenço Chacon

**Affiliations:** 1 Programa de Pós-graduação em Fonoaudiologia, Universidade Estadual Paulista – UNESP - Marília (SP), Brasil.; 2 Programa de Pós-graduação em Estudos Linguísticos, Universidade Estadual Paulista – UNESP - São José do Rio Preto (SP), Brasil.; 3 Departamento de Fonoaudiologia, Faculdade de Filosofia e Ciências, Universidade Estadual Paulista – UNESP - Marília (SP), Brasil.

**Keywords:** Linguagem Infantil, Distúrbio de Linguagem, Ecolalia, Linguística, Fonoaudiologia

## Abstract

Sob ótica linguístico-discursiva de orientação francesa, foram investigadas marcas de subjetividade numa cadeia de enunciados tidos como ecolálicos, ancorados na estrutura linguística recorrente *X quer Y?*. No interior de uma sessão de fonoterapia, essa cadeia foi produzida por J., uma criança do sexo feminino, com 10 anos de idade à época da coleta dos dados, com diagnóstico fonoaudiológico de *distúrbio de linguagem* e diagnóstico médico de *psicose precoce*. Um conjunto de flutuações linguísticas indiciaram um deslizamento de posição subjetiva na cadeia analisada. Tais flutuações envolveram elementos sintáticos, lexicais, semânticos, morfológicos e prosódicos. Discursivamente, as flutuações deixaram rastros de um deslizamento de posição subjetiva, ou seja, de sujeito *falado* (*cê quer Y?*) para *falante/desejante* (*eu quero Y.*) na cadeia formada por esses enunciados. Desse modo, enunciados tidos como ecolálicos podem dar pistas do desejo do sujeito em sua relação com o O/outro, por meio de suas flutuações linguísticas e de seus deslizamentos discursivos. Portanto, embora não irrompam de forma convencional, tais enunciados podem mostrar possibilidades de mudanças de posição subjetiva. Por conseguinte, uma contribuição da investigação relatada à clínica de linguagem é a de que, no *setting* terapêutico, pode haver escuta para enunciados que, em primeira instância, poderiam ser tidos como colados/enraizados no dizer do outro. Na clínica de linguagem é possível, então, dar lugar a novos/outros sentidos a tais enunciados, de maneira a favorecer a constituição do sujeito da/na linguagem a partir de enunciados frequentemente interpretados como esvaziados de subjetividade.

## INTRODUÇÃO

Em contextos patológicos que envolviam, por exemplo, distúrbios de linguagem, as chamadas ecolalias foram inicialmente vistas como “[...] produções desprovidas de sentido, apenas como um eco, semelhante à fala de um papagaio: uma repetição sem intenção comunicativa, sem interlocutores [...] uma repetição vazia, descontextualizada [...].”^(1:416)^. Posteriormente, passaram a ser investigadas sob diferentes abordagens^(2)^.

Predominam abordagens que as relacionam a questões de natureza pragmática, comunicativa ou de interação social^(3-5)^. Mas também, a partir de um escopo enunciativo e multimodal da linguagem, as ecolalias podem ser vistas enquanto metáfora, em sua relação com os gestos no autismo^(1,6)^. Podem, ainda, a partir da relação sujeito/linguagem, ser entendidas como repetições de segmentos sonoros que levariam a mudanças na verbalização^(7)^.

Vê-se, pois – fato que este último estudo permite exemplificar –, que só mais recentemente encontra-se, em sua investigação, um olhar voltado para o *sujeito* que, para além das ecolalias, “fala nessa voz”^(2:2)^, olhar que produz um significativo deslocamento na trajetória de investigação de falas tidas como ecolálicas.

Aproximando-se deste último olhar e afastando-se do lugar teórico-metodológico que as vê como ecos do dizer do(s) outro(s), propôs-se investigar a maneira como tais falas poderiam ser interpretadas sob um ponto de vista linguístico-discursivo de orientação francesa, cujas características serão destacadas mais à frente. Nessa proposta, foram buscadas possíveis marcas de subjetividade em enunciados com estrutura linguística recorrente no funcionamento linguístico-discursivo de J. – uma criança com diagnóstico fonoaudiológico de *distúrbio de linguagem* e diagnóstico médico de *psicose precoce*.

Na expressão *linguístico-discursivo*, o linguístico é entendido como a organização estrutural dos elementos dos diferentes planos da língua. Privilegiou-se, dessa organização, os planos sintático, semântico, morfológico e fonológico (em seu componente prosódico) das falas tidas como ecolálicas. Nessa mesma expressão, o discursivo é entendido como um processo de produção de sentidos, “[...] cuja especificidade reside no tipo de materialidade de sua base, a saber, a materialidade linguística [...].”^(8:179)^, materialidade que se mostra sob forma de enunciados. Destaque-se, porém, que o componente prosódico do plano fonológico da língua, além de manter estreitas relações com os componentes sintático e semântico da língua, indicia, em um processo discursivo, aspectos da subjetividade.

O que se toma aqui por enunciado não é uma unidade estrutural da língua. Em outras palavras, o enunciado não se confunde com a organização sintática de uma sentença ou de uma oração; ele é a sua materialização concreta e única num processo discursivo. Nesse processo, ele irrompe como “[...] um elo da cadeia muito complexa de outros enunciados [...]”^(9:291)^ – o que significa dizer que um enunciado não se inicia em si mesmo, já que se ancora numa rede de enunciados anteriormente produzidos.

Por fim, a concepção de sujeito subjacente à presente abordagem é a de que ele é constituído pelo O/outro, *na/pela linguagem* ^(8,10,11)^. Nessa perspectiva, o sujeito não é o do empirismo, já que não é entendido como a fonte do (seu) discurso, mas como seu suporte e efeito^(10)^. Ainda sobre o sujeito, “[...] é sempre e ao mesmo tempo, sujeito da ideologia e do inconsciente [...] atravessados pela linguagem antes de qualquer cogitação”^(12:188-189)^.

No *Outro* (ideologia e inconsciente) está o lugar "[...] da verdade sobre o sintoma e o desejo"^(13:5)^. Assim, não será negligenciado, na presente investigação, o desejo do sujeito, marcado a partir de deslizamentos no (seu) processo discursivo. Destaque-se, porém, que o Outro, num processo discursivo, vem linguisticamente marcado. Trata-se, nesse caso, da instância que se pode entender como o “outro”, a saber, o indivíduo que, interpelado em sujeito pela linguagem no processo discursivo, se configura como o interlocutor linguisticamente marcado nesse processo. Em síntese, o "outro" corresponde à alteridade que é, de algum modo, mostrada no fio do discurso.

Dentre as características linguístico-discursivas que justificam o interesse pelas marcas de subjetividade no funcionamento de enunciados com estrutura linguística recorrente na criança sob investigação, estão o fato de não se observarem, neles, as marcas linguísticas que mais frequentemente remetem aos indícios de constituição subjetiva observados em crianças sem dificuldades na aquisição da linguagem: a primeira pessoa pronominal, como em *eu*, *meu*, *me*, *mim*, *comigo.* Com efeito, nos enunciados que se esperariam como declarativos de J., observa-se a prevalência pronominal de segunda pessoa (ao invés daquela de primeira pessoa) sob forma interrogativa. Desse modo, enunciados que, convencionalmente, se esperaria emergir no discurso de J. como *quero ir embora*, *quero* dormir, *quero* *água* emergem como *cê quer ir embora? cê quer dormir? cê quer água?*. Eventualmente, a convencional e esperada forma em primeira pessoa de seus enunciados desloca-se para a terceira pessoa – como *a menina quer bolo?* em lugar de *eu quero bolo.*

Nesses enunciados, em termos sintáticos, sua estrutura recorrente é *X quer Y*. Nessa estrutura, o primeiro e o terceiro elemento tanto podem faltar, quanto podem sofrer variações lexicais ao longo da produção discursiva. Mantém-se, porém, como invariantes, as seguintes características linguísticas: semântica, volitiva no verbo *querer*; morfológica, no presente do indicativo desse verbo; e prosódica, no contorno entonacional interrogativo do enunciado. Dada a presença recorrente dessa configuração linguística nos enunciados de J, interroga-se: discursivamente, seria possível observar rastros de um deslizamento de posição subjetiva, ou seja, de sujeito *falado* (*cê quer Y?*) para *falante/desejante* (*eu quero Y.*) na cadeia formada por esses enunciados?. Se sim, como tal deslizamento se efetuaria?

A hipótese subjacente a tal interrogação é a de que a cadeia de enunciados com estrutura linguística recorrente de J. (tradicionalmente chamados de *ecolálicos*) comporia uma sequência na qual os enunciados não seriam apenas “ecos” do dizer do outro, mas uma manifestação/marca de subjetividade na produção discursiva.

Destaque-se que dois estudos se voltaram para a produção discursiva da criança J., buscando, como neste, seus indícios linguístico-discursivos de subjetividade. Em um deles^(14)^, foram as hesitações que se mostraram como tais indícios; já no outro^(15)^, foram marcas de recusa que a indiciaram. Contudo, acredita-se que enunciados com estruturas linguísticas recorrentes, como as que compõem o objeto da presente investigação, poderiam fornecer outros (e diferentes) indícios de subjetividade no discurso de J, ampliando-se a compreensão da maneira como, de forma não convencional, a subjetividade pode ser mostrada em quadros clínicos como os de J.

## APRESENTAÇÃO DO CASO CLÍNICO

A presente investigação foi desenvolvida de acordo com os seguintes procedimentos éticos: parecer da Comissão de Ética em Pesquisa da Faculdade de Filosofia e Ciências da Universidade Estadual Paulista (FFC/UNESP), sob número 0138/2010; e assinatura, pelo responsável, de um Termo de Consentimento Livre e Esclarecido, de acordo com modelo fornecido pelo Centro de Estudos em Educação e Saúde da Faculdade de Filosofia e Ciências da Universidade Estadual Paulista (CEES/UNESP).

Foram utilizados registros – filmado e transcrito – de uma sessão de fonoterapia com cerca de 40 minutos, de uma criança (identificada, nos dados, como J.) do sexo feminino, com 10 anos de idade à época da coleta dos dados. A sessão passou por três procedimentos de transcrição: (i) por um primeiro transcritor; (ii) por uma revisão dessa transcrição por esse mesmo transcritor e pelo supervisor do acompanhamento do percurso clínico da criança; e, por fim, (iii) por um terceiro revisor, que incorporou à transcrição especialmente informações sobre a situação imediata em que se desenvolvia a sessão de terapia. Tais informações incluíam, por exemplo, gestos, expressões faciais e informações prosódicas básicas. A transcrição final foi feita de acordo com as normas propostas para o Projeto de Estudo da Norma Linguística Urbana Culta de São Paulo (Projeto NURC/SP), que investiga o português falado. De acordo com tais normas: **+** corresponde a pausa silenciosa; **?** corresponde à entoação interrogativa; **(( ))** corresponde a comentários do transcritor; **:** corresponde a alongamentos; **/** corresponde a interrupções; **( )** se refere a momentos de fala ininteligível para o transcritor ou a dúvidas na transcrição; e, por fim, **[ ]** corresponde a sobreposição de vozes.

A criança recebeu diagnóstico fonoaudiológico de *distúrbio de linguagem* e diagnóstico médico de *psicose precoce*. À época da coleta dos dados, ela estava, há três anos e meio, em terapia fonoaudiológica. Na condução terapêutica, sustentada por uma visão clínica de orientação pragmática, as sessões visavam a "reestruturação" das manifestações linguísticas da criança. Nessa visão, pautavam-se por estratégias como atividades lúdicas que envolvessem brincadeiras de “faz de conta”, que simulavam situações convencionais do cotidiano nas quais a linguagem compareceria. É o caso, por exemplo, da sessão aqui em análise, baseada na brincadeira de "faz de conta" de dar banho em um bebê.

Sobre o diagnóstico fonoaudiológico de Distúrbio de Linguagem, para além de alterações no plano formal da língua, J. apresentava, principalmente, alterações em aspectos discursivos da linguagem. Quanto a esses aspectos, sobretudo a relação eu/(O)outro se mostrava fragilizada, fato que se podia observar, em sua produção discursiva, por: (i) ausência de primeira pessoa pronominal; (ii) dispersão da cadeia sintagmática; (iii) retorno de enunciados (não necessariamente presentes na cena terapêutica). Dentre esses enunciados, destacam-se aqueles com estrutura linguística recorrente*,* do tipo *X quer Y?*, apresentada anteriormente. Portanto, dado esse destaque, é na/pela irrupção desses enunciados que foram resgatados os indícios de subjetividade que neles se manifestariam.

## DISCUSSÃO

Retomam-se, aqui, as perguntas norteadoras da investigação: discursivamente, seria possível observar rastros de um deslizamento de posição subjetiva, ou seja, de sujeito *falado* (*cê quer Y?*) para *falante* (*eu quero Y.*) na cadeia formada por esses enunciados?. Se sim, como tal deslizamento se efetuaria?

Relembre-se que, na estrutura *X quer Y?*, o primeiro e o terceiro elementos poderiam faltar. Se preenchidos, na posição X, ocorreriam flutuações entre segunda e terceira pessoa, com predomínio da segunda pessoa. Já na posição Y, ocorreriam elementos verbais (como *dormir* ou *escrever*) ou elementos nominais como (*água*). Como se pode observar, faltariam ou variariam os elementos que poderiam ocupar as posições X e Y e se manteriam (i) o elemento volitivo verbal *quer* e (ii) a entonação interrogativa do enunciado.

Apresenta-se, no Quadro 1, um recorte da sessão de terapia que se analisará, no qual, privilegiadamente, irrompem enunciados com essa estrutura. Tais enunciados, no quadro, são destacados em negrito.

**Quadro 1:** Recorte de sessão de terapia com diferentes irrupções de enunciados com estrutura *X quer Y*?

037	**J**	**cê quer dormir?**	J coloca o travesseiro na cama, depois fala olhando para T.
038	T	isso ... eu não vou dormir agora ... cê vai dormir agora?	T arruma o travesseiro na cama e, em seguida, J empurra a cama para o lado.
039	J	((risos))	J encosta a cama na parede.
040	T	eu não vou dormir agora não	J e T se olham.
041	J	senta ... pode sentar	J aproxima-se da cama de brinquedo, e após olhar para T, coloca umas das mãos sobre a cama, como se a apontasse.
042	T	põe o nenê lá pra dormir ... põe	J olha para T, enquanto esta pega a boneca que estava no chão.
043	J	((risos))	J aproxima-se mais da cama.
044	T	ah ...você que quer deitar em cama ... deita aqui ... deixa aí pro nenê ... ah ... não te cabe aí	T, que ainda segura a boneca, aponta para o colchonete, enquanto olha para J. Esta ameaça apoiar-se na cama com os braços.
045	J	ela tá dormindo	J fala olhando para T.
046	T	quem tá dormindo?	J olha para o teto.
047	**J**	**(quer água?)**	T e J se olham.
048	T	nenê tá aqui ó ... comigo	T mostra a boneca em sua mão.
049	**J**	**(quer água?)**	J olha para baixo.
050	T	nenê tá aqui comigo	J pega um brinquedo que estava no chão, pertencente ao kit de banho.
051	**J**	**cê quer isquevrê?**	J fala olhando para T, a qual fica observando a boneca.
052	T	não a gente não vai escrever hoje ... hoje eu não trouxe papel:: pra escrever:: ... nem um lá::pis	Por algum tempo, J permanece olhando para T, depois volta seu olhar para baixo. T fala enquanto começa tirar a roupa da boneca.
053	**J**	**cê quer água?**	J olha para T, enquanto esta continua tirando a roupa da boneca.
054	T	não tô com se::de	J olha para T, enquanto esta continua tirando a roupa da boneca.
055	**J**	(o nenê fez) ... {o nenê fez cocô	J fala olhando para T e aponta o bebê
056	T	{cê vai tomar banho? ... fez cocô? ... vê se tá fedido	J continua olhando para T que, após cheirar a boneca, a oferece para J.
057	J	((J grita))	J pega a boneca da mão de T.
058	T	ahn tá fedi::do	J coloca a boneca na cama.
059	J	**eu vou dormir Mila ... quê que cê tá fazendo?**	J tira o lençol da cama.
060	**T**	tá fedido ... então vamos dar banho ... se o nenê fez cocô	J estende o lençol sobre o colchonete, que está atrás de si.

Observe-se, no quadro, a primeira emergência de um enunciado com a estrutura em questão, a saber: *cê quer dormir?* (enunciado 37). Trata-se daquele que alimentará uma cadeia de enunciados nos quais as flutuações de elementos linguísticos nas posições que acompanham o verbo permitem detectar o deslizamento de posição subjetiva. A cena em análise desenvolveu-se em torno do objeto discursivo *fazer o bebê dormir*. Assim, em grande medida, ela pode ter favorecido a irrupção desse enunciado. Também elementos não verbais podem estar na base de tal irrupção, já que, da cena, constavam uma pequena cama e um pequeno travesseiro.

Mas destaque-se, também, a participação do interlocutor T (o *outro* linguisticamente marcado nesse processo discursivo) na irrupção da cadeia de enunciados com a estrutura linguística em análise. Com efeito, tal enunciado, que poderia ter tido, por parte de T, uma escuta como *eu [J] quero dormir*, foi escutado como *T, cê quer dormir?*, ao que J. recebe como retorno de T.: *isso ... eu não vou dormir agora ... cê vai dormir agora?*. Dada essa não escuta à sua "fala de desejo", J. responde com riso e afastamento da cama. T., por sua vez, mantendo sua não escuta, sugere a J. que a boneca seja colocada para dormir, provavelmente, tentando operacionalizar o cumprimento de seu plano terapêutico – o faz de conta do cuidado rotineiro de um bebê – da perspectiva teórico-clínica que orienta o seu cumprimento, a da abordagem chamada de pragmática.

A não escuta à "fala de desejo" de J., aliada a elementos contextuais do processo discursivo, podem estar na base da irrupção de mais outro dos enunciados com a estrutura aqui em análise: *quer água?* (enunciado 47). Nesse enunciado, ocorre o apagamento da posição *X* e uma flutuação lexical na posição *Y*, de *dormir* (um verbo) para *água* (um substantivo). Irrompe, logo a seguir, nova enunciação do enunciado *quer água?* (enunciado 49). O deslizamento, na produção discursiva, entre *cê quer dormir?* e *quer água?* pode ter se dado, novamente, em função de outra não escuta, por parte de T., para a "fala de desejo" de J. Observa-se, a propósito, que, na cena, T. volta a chamar a atenção para a boneca, dada sua não escuta para o fato de que o enunciado *quer água?* poderia estar ligado, contextualmente, ao cuidado com a boneca. Desse modo, o deslizamento entre os dois enunciados pode ter se dado também em função de elementos não verbais da cena terapêutica. Fazia parte dela uma banheira e um *kit* de banho de brinquedo. Observe-se, a propósito, que, logo em seguida à nova enunciação de *quer água?*, J. pega um brinquedo do chão, pertencente ao *kit* de banho, ação que sugere associação entre esse enunciado e a banheira de brinquedo presente no espaço físico da sessão de terapia – ou, em termos linguísticos, um indício de associação semântica entre *banheira* e *água*.

A continuidade da não escuta à "fala de desejo" de J. faz com que novo enunciado com a mesma estrutura irrompa na cadeia: *cê quer isquevrê?* (enunciado 51). Esse enunciado de J. recebe como retorno, por parte de T., a seguinte resposta: *não a gente não vai escrever hoje ... hoje eu não trouxe papel:: pra escrever:: ... nem um lá::pis*. Observa-se nova flutuação no elemento lexical que preenche a posição Y: de *água* (um substantivo) para *escrever* (um verbo). Destaque-se que, no espaço físico em que a cena se desenvolvia, havia uma mesinha e uma cadeirinha infantil, elementos não verbais que teriam desencadeado conexões com uma atividade de escrita, tal como desenvolvida em contexto escolar.

Mais uma vez, a não escuta por parte de T. parece provocar nova irrupção de enunciado com a mesma estrutura – *cê quer água?* (enunciado 53) –, igualmente com flutuação no preenchimento lexical da posição Y, a saber, de *escrever* (um verbo) para *água* (um substantivo). Trata-se, uma vez mais, de um enunciado que irrompe no processo discursivo como mais uma "fala de desejo" não escutada desse (seu) lugar.

Finalmente, depois de uma sequência de enunciados (53 a 58) em que o objeto discursivo *fazer o bebê dormir* se mostra em conflito com o objeto *dar banho no nenê*, irrompe, no dizer de J., um enunciado que, embora discursivamente conectado à cadeia que vinha se desenvolvendo, rompe a estrutura linguística que sustentava os enunciados que a constituíam: *eu vou dormir Mila ... quê que cê tá fazendo?* É o momento em que, linguística e discursivamente, se mostra o deslizamento de posição subjetiva: de sujeito falado (pelo outro) *cê* a sujeito falante (de si mesmo) *eu*. Sustentam, ainda, esse deslizamento: (i) a flutuação lexical entre o verbo *querer* e a locução verbal *ir dormir*; (ii) a flutuação das características morfológicas do verbo (presente) e da locução (futuro); e, ainda, (iii) a flutuação de suas características semânticas (respectivamente, de volitiva a afirmativa).

Algumas observações devem ser feitas a propósito da irrupção dos enunciados com estrutura linguística *X quer Y?* destacados acima. O percurso do caso clínico da criança mostrou que enunciados com essa estrutura ocorreram em todas as cenas terapêuticas acompanhadas. Destaque-se, ainda, que tais enunciados irromperam mesmo em cenas terapêuticas nas quais aparentemente nenhum objeto físico nelas presente justificaria sua irrupção. Por conseguinte, a emergência de enunciados com estrutura *X quer Y?* tanto pode ter sido mobilizada por elementos verbais e não verbais que ecoavam na cena quanto pelo já-dito/já-vivido que a ancorava, ou, talvez e principalmente, pela conjunção entre o que se desenvolvia na cena em análise e ecos das demais cenas (não somente terapêuticas) que compunham a memória discursiva da criança J.

Reforça-se, pois, como um enunciado não se inicia em si mesmo, mas irrompe como um elo de uma cadeia de enunciados^(9)^ num processo discursivo. Afinal, também um processo discursivo não se inicia em si mesmo, já que se estabelece sobre um discursivo prévio (o interdiscurso), composto, no caso em análise, não apenas do conjunto de cenas terapêuticas entre J e T. mas, também, de múltiplas cenas (não terapêuticas) de que J. era convocada a participar em sua vida diária. Assim, os enunciados que irromperam na cena destacada seriam “[...] a rigor, um efeito do interdiscurso sobre si mesmo [...].”^(8:167)^, o que reforça o fato de que, conforme antecipado, o sujeito não é a fonte do (seu) discurso, mas seu suporte e efeito^(10)^.

Por meio de flutuações entre elementos linguísticos que compõem a estrutura *X quer Y?*, de algum modo relacionadas a elementos verbais e não verbais da cena terapêutica e, como se acabou de destacar, também possivelmente à memória discursiva, a cadeia de enunciados destacada mostrou o deslizamento da posição subjetiva de J. Esse deslizamento não se deu de modo linear/progressivo, como se viu na descrição do recorte. Diferentemente, as flutuações lexicais acompanhavam, por exemplo: (i) desdobramentos não favorecidos no processo discursivo por (muito) pouca negociação de sentidos entre T. e J.; (ii) conflitos de objetos discursivos; e, ainda, (iii) movimentos que mesclavam ecos de outros enunciados da cena e elementos não verbais que a compunham. Trata-se de flutuações que "construíam" o deslizamento discursivo desejado – que, do enunciado de partida ao enunciado de chegada, se pode, linguisticamente, sintetizar por deslocamentos: lexical (*querer / ir dormir*); semântico (volitivo / afirmativo); morfológico (presente / futuro); prosódico (interrogativo / declarativo); e pronominal (eu / você). Nessa conjunção, sobretudo os deslocamentos semântico e pronominal mostram, privilegiadamente, um possível rastro do desejo do sujeito, marcado, em seus pontos extremos, pelo caráter volitivo e afirmativo dos verbos, e pela mudança gramatical de pessoa, ou seja, a explicitação linguística, convencional, da subjetividade.

É o que se vê em *eu vou dormir Mila*, em que as marcas de primeira pessoa apontam o assujeitamento de J. à posição de sujeito falante/desejante, e a continuidade do enunciado *... quê que cê tá fazendo?* possibilita identificar um possível estranhamento da criança em relação à sua própria fala – o que, complementarmente, pode fornecer mais um indício de sua mudança de posição subjetiva. Assim, se vistos sob perspectiva linguístico-discursiva como a assumida no estudo aqui em relato, enunciados tidos como ecolálicos podem mostrar, por sua suposta repetibilidade estrutural (mas não discursiva) um percurso de deslizamento de posição subjetiva.

## COMENTÁRIOS FINAIS

Acredita-se que a investigação relatada tenha fornecido elementos para a interpretação de que uma cadeia de enunciados tidos como ecolálicos (por sua estruturação linguística recorrente) pode, ao mesmo tempo, mostrar rastros de sua ancoragem (i) em elementos contextuais de uma cena terapêutica e (ii) numa rede interdiscursiva que a torna possível. Nessa sua ancoragem, a cadeia pode, ainda, mostrar indícios de subjetividade, se sua irrupção for entendida como uma "fala de desejo" na qual podem ser identificados deslizamentos de posição subjetiva.

No entanto, mostrou-se bastante singular (e complexo) o funcionamento da estrutura *X quer Y?* nas sessões de terapia da criança J. – razão da presente proposta de investigá-lo. Como se pôde observar, as flutuações linguísticas na cadeia de enunciados com estrutura linguística recorrente mostrou que a “[...] deriva [está] sempre pronta a se instalar no discurso, fruto de uma subjetividade fragilmente constituída [...].”^(15:9)^. Mas também o movimento mostrado dessa deriva pode indiciar deslizamentos de posição subjetiva na produção discursiva, uma vez que configura um “[...] lugar logicamente desestabilizado e marcado pela tensão entre o dito e o não-dito, [...]”^(13:27)^.

A presente exposição – acredita-se – pode trazer contribuições para a literatura e para a clínica fonoaudiológica que se volta para questões de linguagem. Como se procurou mostrar, estruturas linguísticas recorrentes podem, nos enunciados por elas sustentados, dar pistas do desejo do sujeito em sua relação com o O/outro, por meio de suas flutuações linguísticas e de seus deslizamentos discursivos. Portanto, embora não irrompam de forma convencional, tais deslizamentos podem mostrar possibilidade de mudanças de posição subjetiva. Por conseguinte, uma contribuição à clínica de linguagem oferecida a partir da investigação de enunciados ancorados por estruturas linguísticas recorrentes pode ser observada, na medida em que, no *setting* terapêutico, pode haver escuta para enunciados que, em primeira instância, poderiam ser tidos como colados/enraizados no dizer do outro. Na clínica de linguagem, é possível, então, dar lugar a novos/outros sentidos a tais enunciados, de maneira a favorecer a constituição do sujeito da/na linguagem a partir de enunciados frequentemente interpretados como esvaziados de subjetividade.

Assim, nos quadros de crianças com distúrbios de linguagem por implicações de natureza psicótica, a Fonoaudiologia pode ir além das análises estruturais/formais e avançar para a significação (verbal e não verbal) propriamente dita. Como se procurou demonstrar, um olhar linguístico-discursivo como aquele aqui proposto, para além de analisar descontextualizadamente expressões com estruturas que poderiam ser interpretadas como ecolálicas, permite analisá-las em relação à complexidade de sua irrupção num processo discursivo.
